# Psychometric evaluation of the german version of the parent-adolescent communication scale

**DOI:** 10.1007/s00787-024-02541-4

**Published:** 2024-08-07

**Authors:** Holger Zapf, Johannes Boettcher

**Affiliations:** Department of Child and Adolescent Psychiatry, Psychosomatics and Psychotherapy, University Medical Center Hamburg-Eppendorf, 20246 Hamburg, Germany

**Keywords:** Parent-adolescent communication, Parent-adolescent relationship, Parent-child relationship, Adolescent mental health, Psychometric properties

## Abstract

The quality of dyadic Parent–Child Communication (PCC) between parents and their adolescent children may be important for the adolescent’s general development and mental health during adolescence. Since German instruments for measuring PCC are still lacking, this study aimed to validate the German translation of the Parent-Adolescent Communication Scale (PACS), an internationally established self-report questionnaire. To examine the theoretical-based two-factor model of the PACS in a German population sample of 1044 parent-adolescent dyads, confirmatory factor analyses were used. Moreover, the cross-group measurement invariance of the instrument was assessed. Two clinical samples were used to assess discriminative validity. Bland–Altman plots were used to asses parent-adolescent agreement. The two-factor structure of the PACS was supported. Scalar measurement invariance was established for parent age groups and adolescent gender but not for adolescent age groups and parent gender. Convergent and divergent validity were established. Discriminative validity was only established for the adolescent version in the adolescent psychiatry sample. Test–retest reliability was satisfactory, and rater agreement was medium. The German translation of the PACS is a reliable and valid measurement to assess parent-adolescent communication quality per self-report. Research directions include the concordance discrepancy in clinical samples and further investigation of changes in parents’ and adolescents’ perspectives on communication problems and open communication. Registration: NCT05332236 (clinicaltrials.gov), first registered 2022-04-11.

**Supplementary Information:**

The online version contains supplementary material available at 10.1007/s00787-024-02541-4.

## Background

The quality of communication within the family is widely acknowledged as an important factor influencing the development and mental health of children and adolescents [45]. In this context, Parent–Child Communication (PCC) could play an important role in the vulnerable developmental period of adolescence [1, 50]. Even though there have been international studies using various instruments to assess PCC in adolescence, there is still no German-language questionnaire that can be used to validly assess the quality of dyadic parent-adolescent communication [57].

PCC can be defined as “the verbal and nonverbal interaction between a parent and child within a family system” [34]. Although this definition conceptualizes PCC technically, it is necessary to clarify the normative dimension of the concept as well as its interpersonal nature [57]. With regard to the normative dimension of PCC quality, Marshall Rosenberg made a major contribution to the concept, claiming that communication should be nonviolent. Nonviolent communication consists of empathetic listening and the honest expression of needs and feelings [42]. If these principles are put into practice, communication will become more open, communication problems will decrease because it is more likely that basic needs will be fulfilled, and, in turn, relationships improve [42]. Vice versa, communication problems and reduced openness indicate lower communication quality. Even though the communication between parents and their adolescent children may appear imbalanced regarding competence and power, Rosenberg stresses that the aforementioned principles also apply to PCC [41].

According to the approach of nonviolent communication, the quality of PCC goes along with the fulfillment of needs. Hence, findings relating it to life satisfaction, the development of a healthy lifestyle, self-esteem, and school competencies of adolescents [23, 24, 29, 47] are in line with theory-based expectations. PCC quality and the resulting improvement in relations is also an important resilience factor in critical life situations: The ability of adolescents to adapt and cope in the context of critical life events has been linked to the quality of PCC in cohorts of parental divorce [48], physical [19] and mental diseases [21, 31], and death [17], as well as physical illness of adolescents [25] and their siblings [16]. Moreover, the adolescent’s mental health also appears to be associated with the quality of PCC, which could be shown for both clinical [37, 56] and non-clinical populations [20, 53]. These findings are also in line with the theory-based assumption that higher quality of PCC indicates a better fulfillment of needs and a better parent-adolescent relationship, which, in turn, improves adolescent mental health [33, 40].

To clarifiy the interpersonal nature of PCC, the Generational Stake Theory [2] is helpful. It posits that parents and children, representing different generations, have distinct psychological needs and varying levels of commitment in forming the generational bond [26]. Therefore, even though the basic principles of communication apply to both parents and adolescents in PCC, both may have different views on the PCC quality, and it should not be expected that parents and adolescents fully agree on the quality of PCC. Adolescents may not feel obliged to disclose the same amount of information their parents believe to be adequate. In contrast to their parents, they may stress autonomy over continuity [27]. From these different stakes, specific problems arise: Adolescents may sometimes face challenges in articulating their thoughts and emotions effectively, while parents may struggle to adapt their communication styles to accommodate their children’s changing developmental needs [28]. This complexity can result in misunderstandings, conflicts, and strained relationships between parents and adolescents and thus can result in poor well-being for both [7].

Therefore, the quality of PCC is of particular importance in research into the etiology of mental disorders, in diagnostics, and in the psychoeducational teaching of disorder models during adolescence. As a result of the numerous social, psychological, and biological changes that manifest during adolescence [9], this developmental stage can be regarded as a pivotal juncture concerning mental health issues [12]. In addition, PCC is a possible starting point for therapeutic and preventive interventions in a wide variety of contexts [57]. The potential benefits of this approach become even more clear when PCC is considered concerning parent–child dyads: Unlike the non-specific assessment of the quality of family communication, which is often part of family functioning questionnaires [6], the assessment of dyadic PCC allows for precise analysis and identification of problematic interactions.

A recent systematic review investigated the psychometric properties of PCC measures and found the Parent–Adolescent Communication Scale (PACS; [4]) to be the most common PCC instrument that has a theoretical foundation, and its psychometric properties have been evaluated in multiple studies [57]. Moreover, the PACS differentiated between clinical and non-clinical populations in previous studies [57]. Although the PACS has been translated and evaluated from English to nine other languages, there is no validated German-language questionnaire instrument to assess dyadic PCC. Therefore, this study aimed to validate the German translation of the PACS, an internationally established questionnaire for the assessment of dyadic communication between parents and their children in different populations.

## Methods

### Procedure

Ethics approval was obtained from the Local Ethics Committee of the Center for Psychosocial Medicine, Hamburg-Eppendorf Medical University Center (LPEK-0396, LPEK-0456). All parents and adolescents provided informed consent. This study was preregistered at ClinicalTrials.gov (NCT05332236).

### Translation of the PACS

The PACS was forward translated from English to German independently by a native English speaker, a psychologist, and a professional translator. A consensus version of the translation was established through discussion. After that, the consensus version was translated back by a native English speaker. A final consensus version was established by discussion and pre-tested in a convenience sample of adolescents and parents.

### Measures

#### Parent-adolescent communication

The quality of dyadic PCC was assessed with the German translation of the Parent-Adolescent Communication Scale (PACS; [4]). This scale was originally designed to add a dynamic component to the Circumplex model of family functioning to explain changes in the curvilinear dimensions of „family cohesion “ and „family adaptability “ as measured by the Family Adaptation and Cohesion Evaluation Scale (FACES) [5, 36]. Since then, it has been used independently in more than 80 studies [57]. The original measure consists of an adolescent and a parent version with 20 identical items that are rated on a scale from 1 (“strongly disagree”) to 5 (“strongly agree”). The subscale ‚open communication ‘ consists of ten items, e.g., „When I ask questions, I get honest answers from my mother/father/child “. The subscale ‚communication problems ‘ consists of ten items, e.g., „My mother/father/child insults me when s/he is angry with me.“ The problem subscale is coded reversely so that higher values indicate better communication quality across all subscales (range: 10–50) and the total scale (20–100). In the original validation study, internal consistency was reported to be α = 0.87 for open communication, α = 0.78 for communication problems, and α = 0.88 for the total scale for the complete sample [4]. The factorial structure found in the original study has been corroborated by the Dutch translation of the PACS [22]. Across later studies, the total scale and the subscales mostly showed adequate to good internal consistencies and discriminated between clinical and non-clinical samples [57].

#### Validation measures

The 28-item Dyadic Relations Scale (DRS) is part of the German version of the Family Assessment Measure [11, 49]. Family members evaluate their perceived relationship with the respective family member (partner/parent/child) of concern. It is assessed on a 4-point Likert scale ranging from 0 = strongly disagree to 3 = strongly agree and entails the subscales task accomplishment, role performance, communication, affective expression, involvement, control, values and norms, and a total scale. Each subscale consists of 4 items. Higher scores indicate more problems in the dyadic relationship. The measure was only administered to adolescents and parents in the normative population sample. The present analysis used only the subscales of communication, control, and values and norms, as the first subscale was expected to show higher convergence with the PACS due to the similar content, whereas the two other subscales were expected to show lower convergence in comparison due to their different content. Internal consistencies for these subscales ranged from α = 0.62 to 0.70.

The Strengths and Difficulties Questionnaire [14] was also administered in all samples. Nevertheless, it is not part of the current analysis.

#### Sociodemographic and clinical variables

In the clinical samples, clinicians completed a study-specific sociodemographic questionnaire about the patient’s sex, age, family and living situation, and clinical diagnoses.

### Data preparation

Complete straightliner (variance = 0 in raw items of questionnaire in parent or adolescent section respectively) and speeder cases (time to complete < 520 s in the population sample) were deleted. Results of separate parent and child straightliners were set to missing values. Reports on referenced attachment figure (mother/father/stepmother/stepfather/foster mother/foster father/other), as well as sex of the attachment figure and age of the child compared to age of attachment figure were checked for plausibility, and inconsistent cases were deleted.

### Samples

This study is based on three samples, with Table 1 showing the main sociodemographic characteristics. The normative population sample was recruited nationwide through an online access panel with the assistance of a demographic consulting company for market and social research (Bilendi). Data were collected between September 2022 and January 2023. Registered adults with children aged ten to 18 years were asked to participate. They were screened for parent sex, parental education, and adolescent sex. Quotas were set for the screening questions to reach a distribution similar to that of the German population [52]. After completing the survey, participants received an incentive in the form of credit points, which they could use together with other collected points to buy goods online. The clinical samples were recruited at the Child and Adolescent Psychiatry (CAP) unit and the Pediatric Surgery (PS) unit of the University Medical Center Hamburg-Eppendorf. Since both mental disorders and physical illnesses in adolescents have been found to affect PCC quality [25, 38, 56], samples were recruited from both sites. The clinical samples were recruited between March 2022 and August 2023. Parents and their adolescent children were asked by clinic staff to complete the survey on paper. The CAP sample included adolescents with psychiatric diagnoses according to the ICD-10. The PS sample included adolescents who had been treated with an abdominal embryonal tumor in the unit of the clinic in the past. Inclusion criteria: Consent to participate. Child age: 10–18 years (clinical samples: 10–17 years, according to the age range treated in the clinic). Exclusion criteria: None (population sample). Acute and severe psychological/medical symptom burden of the adolescent according to clinical appraisal or cognitive impairment (clinical samples). Parents and adolescents both consented to participate in the study and could cancel the survey at any time. Minus the removed cases of straightlining, speeding, and implausible data, 1044 dyads were recruited for the normative population sample. Of these, 1041 parents and 1028 adolescents completed all items. In the CAP sample, 94 parents and 67 adolescents were recruited. In the PS sample, 53 parents and 36 adolescents were recruited.

**Table 1:** Sociodemographic characteristics

Parents	Normative population sample Parent *N* = 1044 Adolescent *N* = 1044		CAP* Parent *N* = 94 Adolescent *N* = 67		PS^*^ Parent *N* = *53* Adolescent *N* = *36*	
	M	SD	M	SD	M	SD
Age (years)	43.6	7.4				
Sex						
Female	42.5	6.9	46.3	5.8	42.92	6.09
Male	45.0	7.9	49.7	6.4	47.19	5.29
Number of children in family	1.9	1.2			2.33	1.21
	n	%	n	%	n	%
Mothers	613	56.9	63	67.0	47	88.7
Step-/foster mothers	19	1.8	2	2.1	–	–
Fathers	431	37.5	31	33.0	27	50.9
Step-/foster fathers	39	3.8	2	2.1	–	–
Marital status						
Married	707	67.7	60	63.8	36	67.9
Living with a partner	128	12.3	5	5.3	8	13.2
Single	111	10.6	10	10.6	3	5.7
Divorced	84	8.0	16	17.0	4	7.5
Widowed	14	1.3	0	0	1	1.9
Not stated			3	3.2	1	1.9
Educational status (mothers/fathers)						
Higher education qualification	412	39.5	28/14	29.8/14.9	31/15	66.0/55.6
General certificate of secondary education	371	35.5	13/4	13.8/4.3	4/5	8.5/18.5
Secondary education	234	22.4	4/4	4.3/4.3	5/4	10.6/14.8
Other	13	1.2	–	–	6/3	12.8/11.1
None	14	1.3	–	–	1/0	2.1/14.8
Not stated	–	–	18/9	19.1/9.6	–	–
Professional qualification (mothers/fathers)						
Vocational training	600	57.5	16/7	17.2/7.4	14/6	29.8/22.2
Master handicraft	143	13.7	1/1	1.1/1.1	4/2	8.5/7.4
University	166	15.9	18/10	19.1/10.6	19/11	40.4/40.7
Other	46	4.4	2/2	2.1/2.1	0/0	0.0/0.0
None	89	8.5	1/0	1.1/0	4/4	8.5/11.1
Not stated			26/11	27.7/11.8	6/4	12.8/14.8
Single parent	215	20.6	–	–	0	0.0
Other language than German at home	37	3.5	4	4.3	0	0.0

Adolescents	M	SD	M	SD	M	SD
Age (years)	13.4	2.2	14.25	2.0	11.62	2.95
	n	%	n	%	n	%
Sex						
Female	524	50.2	34	50.7	30	56.6
Male	512	49.0	28	41.8	23	43.4
Non-binary	8	0.8	4	6.0	0	0.0
Not stated	0	0.0	1	1.5	0	0.0
Primary psychiatric diagnosis (ICD-10)						
F3	–	–	22	32.8	–	–
F4	–	–	19	28.4	–	–
F8	–	–	10	14.9	–	–
F9	–	–	12	17.9	–	–
Other	–	–	4	6.0	–	–
Method of treatment						
Inpatient	–	–	32	47.8	–	–
Outpatient	–	–	35	52.2	–	–

*CAP* child and adolescent psychiatry. *PS* pediatric surgery

*Both mothers and fathers were able to complete the questionnaire in these samples. Hence, the percentages regarding the parents do not add up to 100% in these samples as they represent the portions based on the adolescents

## Statistics

### Statistical analyses

As it was compulsory to answer all items in the population sample to complete the survey, single missing items only occurred in the CAP and PS samples. Different sample sizes resulted if parents or adolescents did not complete all items or were identified as straightliners (variance of original item values = 0). In the CAP and PS samples, the complete randomness of missing data was tested. If less than 5 of 10 items per original subscale were missing, the scale was computed by multiplying the mean of the remaining items with the number of items to increase the scale’s clinical utility. Sensitivity analyses were conducted to test whether the results of cases with complete items and cases with < 5 missing items differed. In the factor analyses, missing data were excluded listwise.

Initially, an item analysis including item difficulty, range, and corrected item-total correlations was conducted. Items that did not cover the whole range and showed item difficulty P < 20 or > 80 were considered for removal, as well as items with an item-rest correlation < 0.4. Model fit of the original scale was tested in a confirmatory factor analysis. The estimator for ordinal data was DWLS [30]. Indices for model fit were Comparative Fit Index (CFI), Tucker-Lewis Index (TLI), Root Mean Square Error of Approximation (RMSEA), and Standardized Root Mean Square Residual (SRMR) [18]. The χ^2^ test statistic was reported but considered only as additional information because it tends to reject models in large samples [8]. A ratio of χ2/df (degrees of freedom) < 2 indicates a superior model fit [3]. Thresholds for good model fit were > 0.95 for CFI and TLI (> 0.90 is acceptable, RMSEA < 0.06 (< 0.09 is acceptable, and SRMR < 0.08. Measurement invariance was tested for male and female respondents (both parents and adolescents and different age groups (adolescents: 10–13, 14–18,parents: ≤ 43, ≥ 44, median split according to [15]. Three hierarchical levels of measurement invariance were tested: Configural, metric, and scalar. The threshold for the *p*-value in model comparisons was set to *p* > 0.05. Model comparisons were only conducted if the previous model had a sufficient fit. If measurement invariance was not established, partial measurement invariance was aimed for in the process of successively setting significant parameters free.

Internal consistencies of the resulting subscales and the total scale were calculated. Reliability was assessed using Cronbach’s α with Alpha > 0.90 as very good, > 0.80 as good, and > 0.70 as acceptable. A random subsample assessed test–retest reliability 12 weeks after the initial survey.

To assess convergent validity, correlation with the DRS subscale communication was examined. To assess divergent validity, correlation with the DRS subscale control and values and norms was examined. Both constructs are expected to be associated with parent-adolescent communication, but since they represent different constructs, they were expected to show weaker associations with PCC than DRS communication. All correlations were calculated as Pearson’s *r*.

A mixed model for each scale was calculated for the general population, the CAP, and the PS sample to assess discriminative validity. A sample size calculation prior to the start of data collection resulted in a sample size of N = 1000 for the population sample and N = 33 for each clinical sample with a power of 80% and a medium effect (with alpha level = 0.05). Fixed effects were sample, adolescent and parent gender, adolescent age, and parental education. The family code was specified as a random effect. We decided to consider mean group differences > 10% of the scale range as clinically relevant, i.e., differences > 8 raw points on the total scale (with a range of 80) and > four raw points on the subscales (with a range of 40).

Correlations and Bland–Altman plots were used to assess the agreement between parent and adolescent dyads for the subscales as well as the total scale [13]. It is important to note that PCC is not a single subject. Instead, adolescents and their parents may have different perceptions of the quality of communication, so we expected only medium correlations. In the Bland–Altman plots, we expected a distribution parallel to the horizontal line, indicating that parent and adolescent perceptions of communication quality do not differ more at lower values. Since this analysis was exploratory and none of the rater perspectives can be interpreted as a gold standard, we did not prespecify limits of agreement for the Bland–Altman plots.

To develop a possible short form, three items per subscale were selected considering item content, item difficulty, item-rest correlation, factor loadings in the CFA, and problematic items in the measurement invariance analysis. A correlation with the original scale would be overestimated when using the same sample; this can only be considered a first step in developing a short form [32, 51]. To assess the psychometric properties of the short form, validation in a new and independent sample is necessary [51]. The statistical analyses were conducted in JASP version 0.17.3 and R version 4.3.2.

## Results

### Item analysis

In the adolescent form, all items showed values in the full range of the scale and an item-total correlation > 0.4. Items 7, 8, 13, and 19 were above the threshold for item difficulty. In the parent form, all items showed values in the full range of the scale and an item-total correlation > 0.4. Items 1, 9, and 18 were above the threshold for item difficulty.

### Factorial structure

**Fig. 1:**
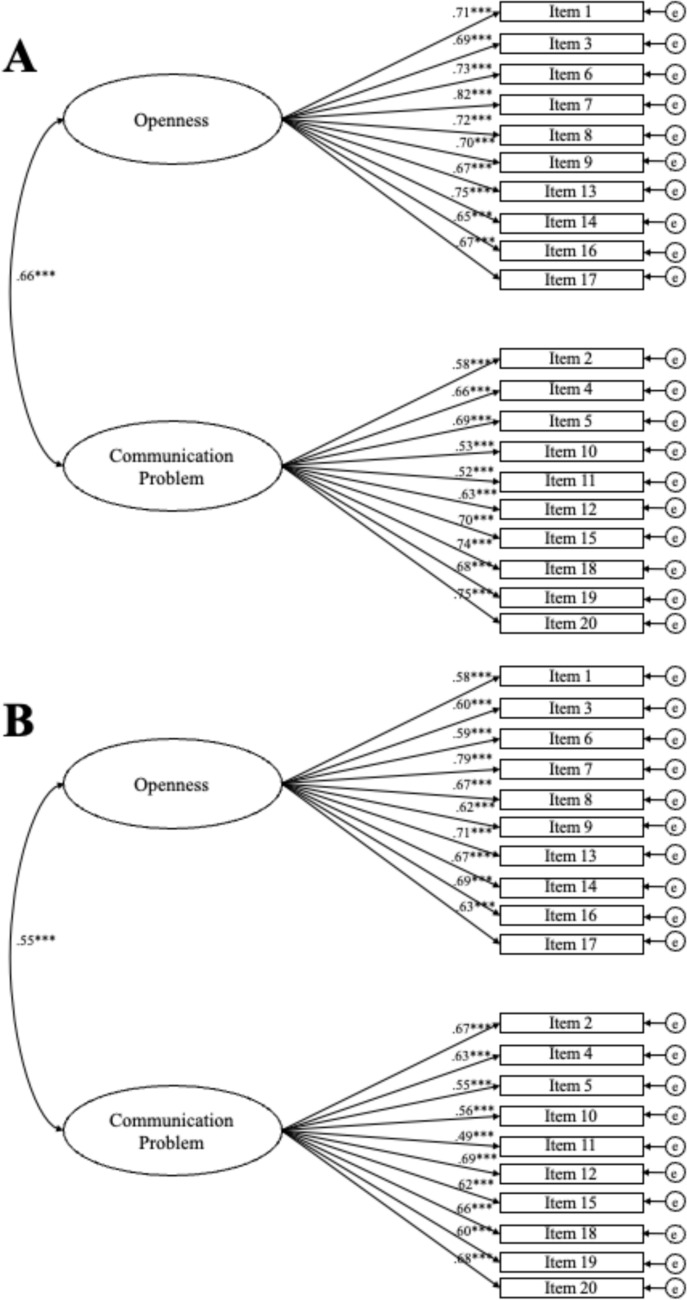
Standardized factor loadings and factor correlations of the PACS. Note. ***p < .001

We tested whether the data fit the structure of the original English version of the scale with two factors [4]. As shown in Table 2, indices were above the thresholds for good model fit in the adolescent and parent versions of CFI, TLI, RMSEA, and SRMR. As expected for a sample of this size, the χ^2^-test yielded a significant result. However, the ratio χ^2^/df indicates a superior model fit in the adolescent sample and an acceptable fit in the parent sample. The CFA factor loadings for parents and adolescents of the final model are displayed in Fig. 1.

**Table 2:** General model fit PACS

Model	χ^2^	df	*p*	χ^2^/df	CFI	TLI	RMSEA [90% CI]	SRMR
Adolescent reports								
Original model (20 items, 2 factors)	314.9	169	< .001	1.86	.99	.99	.03 [.02-.05]	.04
Parent reports								
Original model (20 items, 2 factors)	487.3	169	< .001	2.88	.98	.98	.04 [.04-.05]	.05

### Measurement invariance

In the adolescent sample, metric, but not scalar measurement invariance, was established for male and female respondents, as shown in Table 3. Partial scalar measurement invariance could be established in an adjusted model after refitting the model with free intercept parameters for items 9 and 10. For respondents aged 10–13 and 14–18, only configural measurement invariance could be established. Refitting the model for parameters significantly influencing the model fit did not yield sufficient improvement to establish partial metric measurement invariance. In the parent sample, metric, but not scalar measurement invariance, was established for male and female respondents. Refitting the model for parameters that significantly influence the model fit did not yield sufficient improvement to establish partial scalar measurement invariance. For respondents aged ≤ 43 and ≥ 44, scalar measurement invariance could be established.

**Table 3:** Parent-Adolescent Communication Scale (model fit summary)

Model/Groups	Overall fit indices				Comparative fit indices			
Adolescents	${\upchi }^{2}$	df	RMSEA	CFI	Model comparisons	$\Delta {\upchi }^{2}$	$\Delta \text{CFI}$	*p*
Sex								
Configural^1^	409.20	338	.020	.997	–	–	–	–
Metric^2^	458.66	356	.024	.995	1 vs. 2	15.096	.003	.655
Scalar^3^	477.79	374	.023	.995	2 vs. 3	34.701	.001	.010
Scalar adj	466.25	372	.037	.988	2 vs. 3 adjusted	14.338	.001	.574
Age groups								
Configural^1^	376.94	338	.015	.998	–	–	–	–
Metric^2^	503.05	356	.028	.993	1 vs. 2	37.375	.003	< .001
Parents	${\upchi }^{2}$	df	RMSEA	CFI	Model comparisons	$\Delta {\upchi }^{2}$	$\Delta \text{CFI}$	*p*
Sex								
Configural^1^	568.20	338	.036	.985	–			
Metric^2^	648.42	356	.040	.981	1 vs. 2	25.111	.003	.097
Scalar^3^	704.37	374	.041	.978	2 vs. 3	89.380	.002	< .001
Age groups								
Configural^1^	581.93	338	.037	.984	–			
Metric^2^	645.24	356	.040	.981	1 vs. 2	20.508	.003	.305
Scalar^3^	660.65	374	.038	.981	2 vs. 3	25.096	.001	.122

*RMSEA* root mean square error of approximation, cutoff = .06; *CFI* comparative fit index, cutoff = .95

### Internal consistency

Cronbach’s α ranged from α = 0.92 to α = 0.67 across all samples (Table 4).

**Table 4:** Internal consistencies, test–retest-reliability, and convergent and divergent validity

	Parents			Adolescents		
	Open	Problems	Total	Open	Problems	Total
Internal consistency	α	α	α	α	α	α
General Population sample	.88	.86	.90	.91	.88	.92
Child and Adolescent Psychiatry	.83	.75	.84	.91	.81	.90
Pediatric Surgery	.87	.67	.83	.81	.70	.80
Reliability and validity	*r* [95%-CI]	*r* [95% -CI]	*r* [95% -CI]	*r* [95% -CI]	*r* [95% -CI]	*r* [95% -CI]
Test–retest reliability*	.77 [.68, .84]	.64 [.49, .78]	.73 [.62, .82]	.77 [.67, .84]	.56 [.40, .71]	.70 [.59, .78]
Convergent validity: correlation with DRS subscale communication*	− .67 [− .64, − .71]	− .65 [− .62, − .69]	− .77 [− .75, − .80]	− .69 [− .65, − .72]	− .73 [− .70, − .76]	− .80 [− .77, − .82]
Divergent validity: correlation with DRS subscale norms & values*	− .64 [− .60, − .68]	− .46 [− .41, − .51]	− .64[− .60, − .68]	− .69 [− .65, − .73]	− .57[− .53, − .61]	− .70 [− .67, − .74]
Divergent validity: correlation with DRS subscale control*	− .48 [− .43, − .53]	− .38 [− .32, − .43]	− .50 [− .44, − .54]	− .47 [− .41, − .52]	− .37 [− .32, − .43]	− .47 [− .42, − .52]

The 95% confidence intervals of the correlations are shown in brackets

*Investigations within the general population sample group

### Test–retest reliability

In the population sample, a random subsample answered the PACS again after 12 weeks to assess test–retest reliability. It ranged from *r* = 0.77 to r = 0.56 (Table 4).

### Convergent and divergent validity

The correlation of the PACS with the DRS subscale communication was higher than with the DRS subscale control through all subscales and respondents and higher than with the DRS subscale values and norms through all but one subscale for adolescents, in which it was similar (Table 4).

### Discriminative validity

After controlling for adolescent age, parent and adolescent gender, and parental education, mixed models showed relevant group differences only for the adolescent open communication subscale and total scale (Table 5). On average, adolescents from the CAP sample reached clinically relevant worse open and total values compared to the population and the PS sample. Adolescents from the PS sample reached slightly better values on average than adolescents from the population sample. In the parent sample, there were no clinically relevant group differences. Parents from the PS sample reached the highest values on average across all scales. The means and standard deviations of all samples are shown in Supplementary Table 1.

**Table 5:** Mean differences of raw values between groups

Comparison	Parents			Adolescents		
	Open	Problems	Total	Open	Problems	Total
CAP vs. population	− 0.57 [− 2.48, 1.34] *p* = .49	0.80 [− 1.38, 2.97] *p* = .39	0.25 [− 3.25, 3.75] *p* = .87	− 5.73 [− 7.66, -3.81] *p* < .001	− 3.54 [− 5.89, − 1.19] *p* < .001	− 9.21 [− 13.01, − 5.41] *p* < .001
PS vs. population	1.94 [− 0.71, 4.59] *p* = .09	2.80 [− 0.22, 5.83] *p* = .03	4.68 [− 0.18, 9.54] *p* = .03	0.35 [− 3.03, 3.72] *p* = .24	− 0.19 [− 3.97, 3.59] *p* = .91	0.13 [− 6.25, 6.50] *p* = .96
PS vs. CAP	2.51 [− 0.69, 5.70] *p* = .07	2.00 [− 1.64, 5.65] *p* = .20	4.43 [− 1.43, 10.30] *p* = .08	6.08 [2.26, 10.00] *p* < .001	3.35 [− 1.03, 7.72] *p* = .08	9.34 [2.05, 16.63] *p* = .003

### Rater agreement

The correlations between adolescent and parent ratings were *r* = 0.67 (open), 0.69 (problems), and 0.74 (total scale). Visual inspection of the Bland–Altman plots showed a distribution parallel to the horizontal line. Extreme differences between parent and adolescent ratings are rare and more likely to occur at lower mean values (see Supplementary Fig. 1).

### Short form

Considering item content, item difficulty, item-rest correlation, factor loadings, and problematic items in the analysis of measurement invariance, items 7, 14, and 16 from the open and items 4, 12, and 19 from the communication problems subscale were identified as a possible short form of the instrument.

## Discussion

It is well established that the quality of communication within the family plays an essential role in adolescents’ development and mental health [45]. In this regard, psychological research could benefit from well-validated and psychometrically robust instruments that measure intra-family communication. The current study sought to evaluate the psychometric properties of the German version of the PACS in a large population sample and two clinical samples.

### Factor structure and measurement invariance

The original structure with the subscales of open communication and communication problems fit the data very well in the case of the adolescents and the parents and therefore confirmed the initially proposed two-factor model of the PACS [4]. However, (partial) scalar measurement invariance could only be established for parents across age groups and adolescents across gender groups. The means of the scale can be compared between these groups. For the adolescent scale, only configural invariance could be established for the age groups 10–13 and 14–18. These findings may indicate that common factors are associated with similar items across groups while factor loadings differ, and means may not be compared [15]. In other words, the meaning of the items changes during adolescence. It is noteworthy that the two age groups were constructed arbitrarily. It is possible that metric or scalar measurement invariance can be established if age thresholds are set differently. Nevertheless, testing measurement invariance for age, especially for parental age, is crucial to ensuring that the PACS consistently measures the construct across different age groups, demonstrating that PCC has the same meaning to these groups [39]. In the case of the parent scale, metric measurement invariance could be established across gender groups. This indicates that factor loadings are similar for mothers and fathers, while means should not be compared between them. To our knowledge, measurement invariance of the PACS between these groups has not yet been tested in other studies.

### Item analysis

All items of the adolescent and the parent scale showed good properties. However, some were above the threshold for item difficulty. For clinical reasons, we have decided not to remove these items as they still showed values in the full range of the scale and can, therefore, indicate communication patterns that are particularly strained. A short version of the scale should not include more than one of these items to avoid a deviation from the normal distribution that is too great.

### Internal consistency

In the population sample, internal consistency was good to excellent across scales and the total scale, and the open communication subscale were good to excellent across all samples. The findings regarding internal consistency are in line with previous research [57]. In the clinical samples, the internal consistency of the communication problems subscale was still acceptable but reached the lowest values. This may reflect the fact that communication problems are manifold, and items mirror very specific problems in communication that are not necessarily related to other problems in communication. For example, giving the other person a silent treatment when there is a problem and insulting the person are possible indicators of problem communication. Both kinds of behavior may appear independently from each other and sometimes even exclude each other. Higher values for the internal consistency of the problem subscale in the population sample may result from fewer communication problems in this sample.

### Test–retest reliability

The test–retest reliability after twelve weeks was solely medium across all scales, with the lowest value in the communication problems subscale. These findings on the test–retest reliability are consistent with previous research on the PACS [44, 57]. Indeed, the perceived quality of PCC can change over weeks. This result could be explained by the cognitive phenomenon of *Response Shift Theory* [46], in which a person’s evaluation of a construct changes over time. Therefore, the perception may be influenced by recent interactions and communication problems. Considering these results, the instruction for the scale should be amended by a specific time interval that the respondents should think of when answering the instrument. Moreover, future research should use current methods for detecting response shifts (see [46]).

### Convergent and divergent validity

Compared to the DRS subscales communication, control, and values and norms, the highest correlations were with the communication subscale, indicating that the PACS measures something similar. These findings are in line with previous research on the PACS [57].

### Discriminative validity

Regarding discriminative validity, the PACS differentiated clearly between the adolescents from the CAP sample and the adolescents from the other samples. This is in line with previous research [37, 55]. However, in contrast to previous results, open communication and not communication problems have been the subscale that differentiates best. This may be an effect of the CAP sample, indicating that parents in this sample have, on average, high resources when dealing with their children. In contrast to previous research [37, 55], parents in the CAP sample did not differ relevantly from the population sample when rating communication quality with their child. This, as well, may indicate that the parents in the CAP sample have relatively high interpersonal competencies and may not be representative of parents of adolescents with a psychiatric condition in general.

### Rater agreement

The agreement of parents and adolescents was medium. This partial concordance of the dyadic PCC is in line with previous research [54] and the *Generational Stake Theory* [2]. It may be a normative aspect of adolescence that children and their parents have different views on their relationship, with parents expressing more positive views than adolescents (Kapetanovic & Boson, 2022). This may also partly explain the high values of the parents compared to their children in the CAP sample.

## Strengths and limitations

To our knowledge, this is the first study to assess the measurement invariance of the PACS and the rater agreement based on Bland–Altman plots. Moreover, the study was pre-registered in an international registry prior to the start of data collection and was conducted with a sufficiently large sample of adolescents and parents from the main educational groups. It provides important information about the expected rater agreement and the limits of comparability between groups. While the findings of the current study contribute valuable insight, there are limitations to consider. Firstly, while the clinical samples included parent-adolescent dyads from the CAP and PS samples, the normative population sample comprised participants through an online access panel, which may underlie sampling bias and, therefore, might limit the generalizability, which is often present in the development research literature [35]. Moreover, despite the instructions in the online survey, the parent or the adolescent may have been present while filling in the survey, which may have led to increased socially desirable response behavior. Therefore, the concordance of PCC ratings between adolescents and parents could be overestimated. Furthermore, self-report measures were used solely in the study, and the cross-sectional study design may have been susceptible to measuring bias [43]. Furthermore, despite the advantage of having used a dyadic instrument for PCC, other factors such as stress, mentalization ability, and ability to regulate emotions may be important to consider [10].

## Conclusion

The current study’s findings suggest that the German Version of the PACS is a reliable measure for assessing dyadic PCC. However, measurement invariance could be established only for some groups, indicating that comparisons between groups should be conducted carefully as they might be flawed. The multi-perspective assessment of dyadic PCC may be an essential measure for clinicians and researchers to identify associations with mental health issues in adolescents due to dysfunctional interfamilial communication [45]. Future research should further investigate the discrepancy in the perception of communication quality between parents and adolescents that was found in the CAP sample. Also, a short form of the measure should be established. In addition, longitudinal research should be conducted with several measurements within a short period to understand whether the perception of communication problems is more likely to change due to short-term difficulties in interaction than open communication. On this basis, it will be easier to decide whether future longitudinal studies should be based on the concept of open communication or of communication problems, and the effect of clinical interventions on parent-adolescent communication will become clearer.

## Supplementary Information

Below is the link to the electronic supplementary material.

Supplementary file1 (DOCX 828 KB)

Supplementary file2 (PDF 88 KB)

Supplementary file3 (DOCX 24 KB)

## Data Availability

The datasets generated during the current study are available from the corresponding author upon reasonable request.
