# Supplementary material for: Psychometric evaluation of the german version of the parent-adolescent communication scale
Source: Eur Child Adolesc Psychiatry. 2024 Aug 7;34(3):1097–109. doi: 10.1007/s00787-024-02541-4 (PMC11909066; doi:10.1007/s00787-024-02541-4)

**Supplementary Figure 1.** Bland-Altman Plots

**Supplementary Figure 1A.** Bland-Altman plot of agreement between parents and adolescents for PACS Open communication


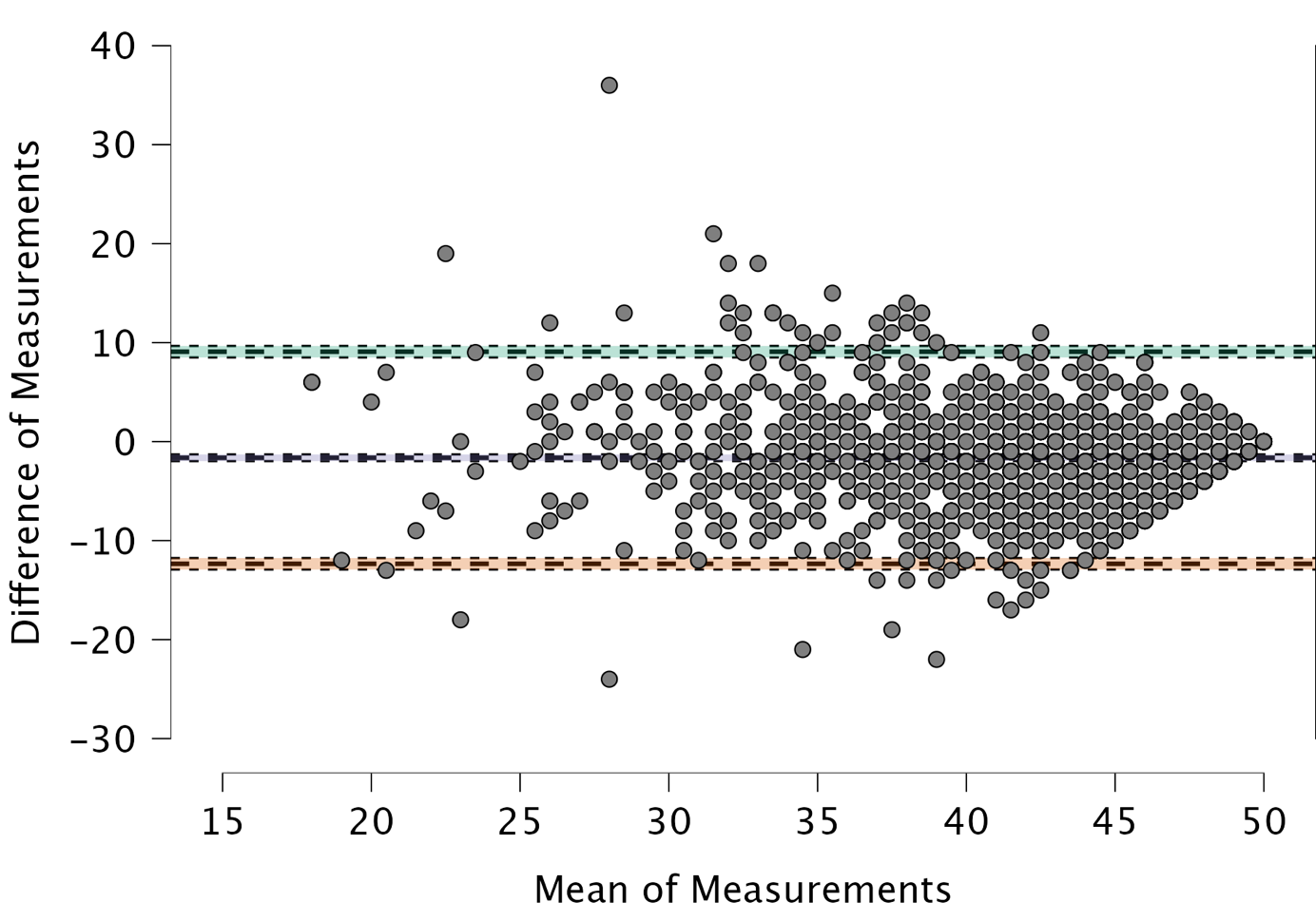


**Supplementary Figure 1B.** Figure Bland-Altman plot of agreement between parents and adolescents for PACS problem communication


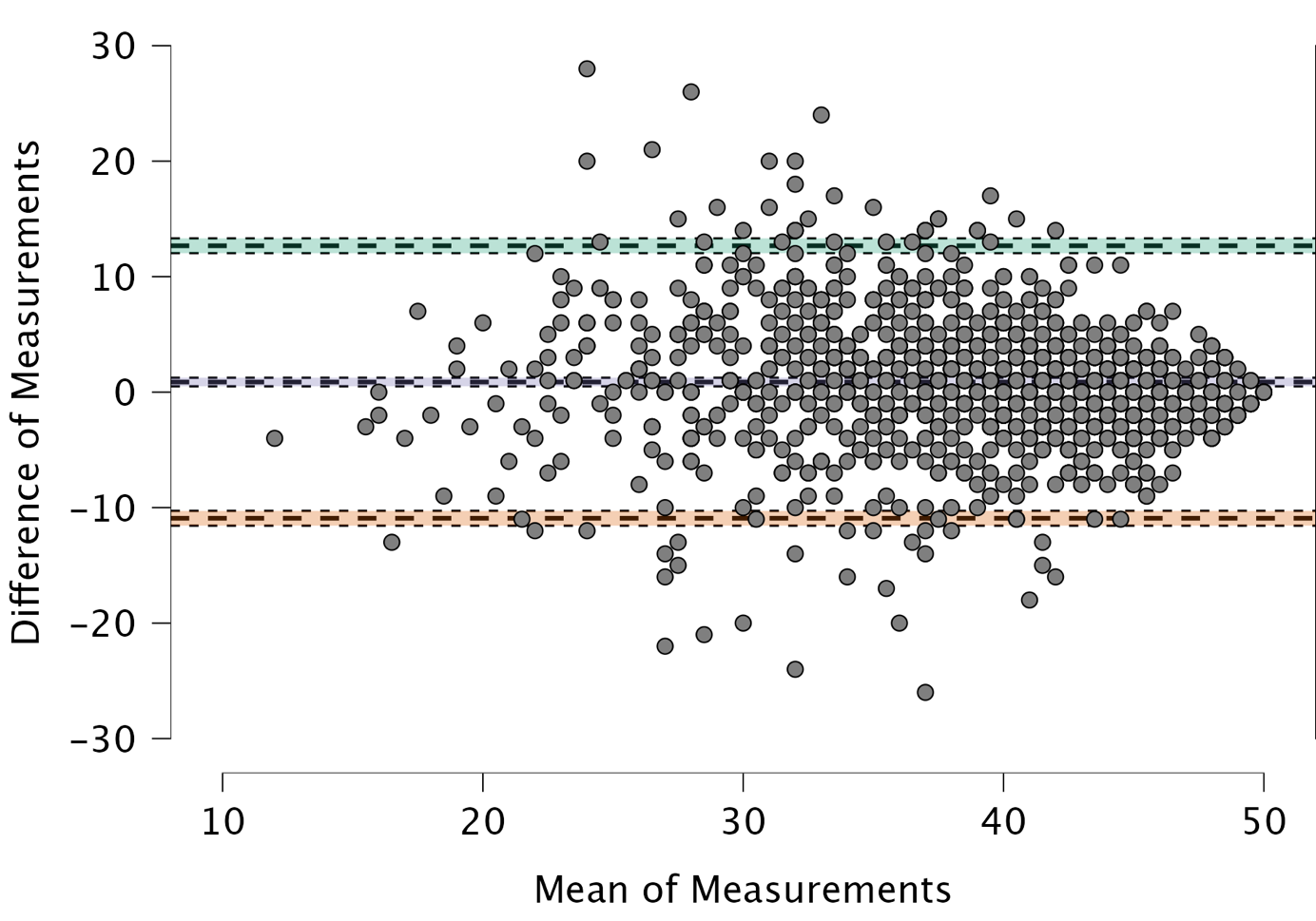


**Supplementary Figure 1C.** Figure Bland-Altman plot of agreement between parents and adolescents for PACS Total


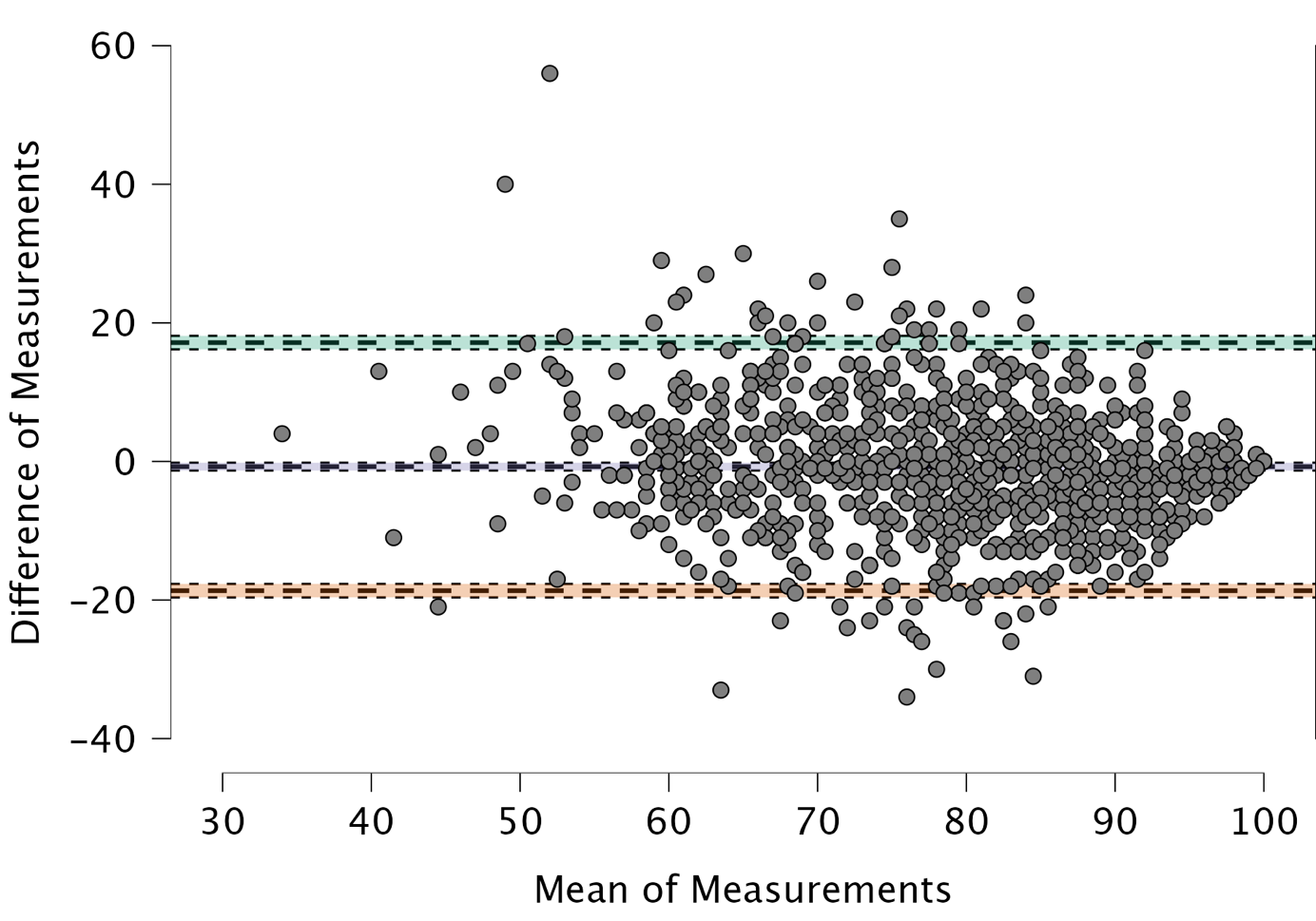

Supplement: Supplementary file 1 — Supplementary file1 (DOCX 828 KB) [file 787_2024_2541_MOESM1_ESM.docx]
