# Supplementary material for: Psychometric evaluation of the german version of the parent-adolescent communication scale
Source: Eur Child Adolesc Psychiatry. 2024 Aug 7;34(3):1097–109. doi: 10.1007/s00787-024-02541-4 (PMC11909066; doi:10.1007/s00787-024-02541-4)
Supplement: Supplementary file 2 — Supplementary file2 (PDF 88 KB) [file 787_2024_2541_MOESM2_ESM.pdf]

Psychometric Evaluation of the German version of the Parent-Adolescent Communication Scale (PACS)

Online Supplementary Material:

German version of the PACS

Holger Zapf, Johannes Boettcher

- Es folgen einige Fragen zur Kommunikation zwischen Dir und Deiner Mutter/Sorgeberechtigten.
- Bitte beantworte die Fragen so gut Du kannst.
- Wenn keine Antwortmöglichkeit genau passt, wähle bitte die am ehesten passende Antwortmöglichkeit.

	Bitte gib hier an, für wen Du diesen Fragebogen ausfüllst:<br>□ 1 **Mutter** □ 2 **Andere**, und zwar: ___________	Trifft voll-kommen zu	Trifft über-wiegend zu	Trifft teilweise zu	Trifft kaum zu	Trifft über-haupt nicht zu
1.	Ich kann meine Ansichten mit meiner Mutter besprechen, ohne mich gehemmt oder verlegen zu fühlen.	□ 5	□ 4	□ 3	□ 2	□ 1
2.	Manchmal fällt es mir schwer, alles zu glauben, was meine Mutter mir erzählt.	□ 5	□ 4	□ 3	□ 2	□ 1
3.	Meine Mutter ist immer eine gute Zuhörerin.	□ 5	□ 4	□ 3	□ 2	□ 1
4.	Manchmal traue ich mich nicht, meiner Mutter zu sagen, was ich möchte.	□ 5	□ 4	□ 3	□ 2	□ 1
5.	Meine Mutter neigt dazu, mir Dinge zu sagen, die besser ungesagt bleiben sollten.	□ 5	□ 4	□ 3	□ 2	□ 1
6.	Meine Mutter weiß, wie ich mich fühle, ohne mich zu fragen.	□ 5	□ 4	□ 3	□ 2	□ 1
7.	Ich bin sehr zufrieden damit, wie meine Mutter und ich miteinander sprechen.	□ 5	□ 4	□ 3	□ 2	□ 1
8.	Wenn ich in Schwierigkeiten wäre, könnte ich das meiner Mutter erzählen.	□ 5	□ 4	□ 3	□ 2	□ 1
9.	Ich zeige meiner Mutter offen Zuneigung.	□ 5	□ 4	□ 3	□ 2	□ 1
10.	Wenn wir ein Problem miteinander haben, strafe ich meine Mutter oft mit Schweigen.	□ 5	□ 4	□ 3	□ 2	□ 1
11.	Ich bin vorsichtig damit, was ich zu meiner Mutter sage.	□ 5	□ 4	□ 3	□ 2	□ 1
12.	Wenn ich mit meiner Mutter spreche, neige ich dazu Dinge zu sagen, die besser ungesagt bleiben sollten.	□ 5	□ 4	□ 3	□ 2	□ 1
13.	Wenn ich Fragen stelle, bekomme ich ehrliche Antworten von meine Mutter.	□ 5	□ 4	□ 3	□ 2	□ 1
14.	Meine Mutter versucht meine Sichtweise zu verstehen.	□ 5	□ 4	□ 3	□ 2	□ 1
15.	Ich vermeide es, manche Themen mit meiner Mutter zu besprechen.	□ 5	□ 4	□ 3	□ 2	□ 1
16.	Es fällt mir leicht, Probleme mit meiner Mutter zu besprechen.	□ 5	□ 4	□ 3	□ 2	□ 1
17.	Es fällt mir sehr leicht, gegenüber meiner Mutter alle meine Gefühle aufrichtig auszudrücken.	□ 5	□ 4	□ 3	□ 2	□ 1
18.	Meine Mutter stört/nervt mich.	□ 5	□ 4	□ 3	□ 2	□ 1
19.	Meine Mutter beleidigt mich manchmal, wenn sie wütend auf mich ist.	□ 5	□ 4	□ 3	□ 2	□ 1
20.	Ich glaube ich kann meiner Mutter nicht sagen, was ich wirklich über manche Dinge denke.	□ 5	□ 4	□ 3	□ 2	□ 1

- Es folgen einige Fragen zur Kommunikation zwischen Dir und Deinem Vater/Sorgeberechtigten.
- Bitte beantworte die Fragen so gut Du kannst.
- Wenn keine Antwortmöglichkeit genau passt, wähle bitte die am ehesten passende Antwortmöglichkeit.

	Bitte gib hier an, für wen Du diesen Fragebogen ausfüllst:<br>□ 1 **Vater** □ 2 **Andere**, und zwar: ___________	Trifft voll-kommen zu	Trifft über-wiegend zu	Trifft teilweise zu	Trifft kaum zu	Trifft über-haupt nicht zu
1.	Ich kann meine Ansichten mit meinem Vater besprechen, ohne mich gehemmt oder verlegen zu fühlen.	□ 5	□ 4	□ 3	□ 2	□ 1
2.	Manchmal fällt es mir schwer, alles zu glauben, was mein Vater mir erzählt.	□ 5	□ 4	□ 3	□ 2	□ 1
3.	Mein Vater ist immer eine guter Zuhörer.	□ 5	□ 4	□ 3	□ 2	□ 1
4.	Manchmal traue ich mich nicht, meinem Vater zu sagen, was ich möchte.	□ 5	□ 4	□ 3	□ 2	□ 1
5.	Mein Vater neigt dazu, mir Dinge zu sagen, die besser ungesagt bleiben sollten.	□ 5	□ 4	□ 3	□ 2	□ 1
6.	Mein Vater weiß, wie ich mich fühle, ohne mich zu fragen.	□ 5	□ 4	□ 3	□ 2	□ 1
7.	Ich bin sehr zufrieden damit, wie mein Vater und ich miteinander sprechen.	□ 5	□ 4	□ 3	□ 2	□ 1
8.	Wenn ich in Schwierigkeiten wäre, könnte ich das meinem Vater erzählen	□ 5	□ 4	□ 3	□ 2	□ 1
9.	Ich zeige meinem Vater offen Zuneigung.	□ 5	□ 4	□ 3	□ 2	□ 1
10.	Wenn wir ein Problem miteinander haben, strafe ich meinen Vater oft mit Schweigen.	□ 5	□ 4	□ 3	□ 2	□ 1
11.	Ich bin vorsichtig damit, was ich zu meinem Vater sage.	□ 5	□ 4	□ 3	□ 2	□ 1
12.	Wenn ich mit meinem Vater spreche, neige ich dazu Dinge zu sagen, die besser ungesagt bleiben sollten.	□ 5	□ 4	□ 3	□ 2	□ 1
13.	Wenn ich Fragen stelle, bekomme ich ehrliche Antworten von meinen Vater.	□ 5	□ 4	□ 3	□ 2	□ 1
14.	Mein Vater versucht meine Sichtweise zu verstehen.	□ 5	□ 4	□ 3	□ 2	□ 1
15.	Ich vermeide es, manche Themen mit meinem Vater zu besprechen.	□ 5	□ 4	□ 3	□ 2	□ 1
16.	Es fällt mir leicht, Probleme mit meinem Vater zu besprechen.	□ 5	□ 4	□ 3	□ 2	□ 1
17.	Es fällt mir sehr leicht, gegenüber meinem Vater alle meine Gefühle aufrichtig auszudrücken.	□ 5	□ 4	□ 3	□ 2	□ 1
18.	Mein Vater stört/nervt mich.	□ 5	□ 4	□ 3	□ 2	□ 1
19.	Mein Vater beleidigt mich manchmal, wenn er wütend auf mich ist.	□ 5	□ 4	□ 3	□ 2	□ 1
20.	Ich glaube ich kann meinem Vater nicht sagen, was ich wirklich über manche Dinge denke.	□ 5	□ 4	□ 3	□ 2	□ 1

Es folgen einige Fragen zur Kommunikation zwischen Ihnen und Ihrem Kind. Bitte beantworten Sie die Fragen so gut Sie können. Wenn keine Antwortmöglichkeit genau passt, wählen Sie bitte die am ehesten passende Antwortmöglichkeit.

	Bitte geben Sie hier an das Alter des Kindes an, für das Sie diesen Fragebogen ausfüllen:<br><br>Alter des Kindes: ________	Trifft voll-kommen zu	Trifft über-wiegend zu	Trifft teilweise zu	Trifft kaum zu	Trifft über-haupt nicht zu
1.	Ich kann meine Ansichten mit meinem Kind besprechen, ohne mich gehemmt oder verlegen zu fühlen.	☐ 5	☐ 4	☐ 3	☐ 2	☐ 1
2.	Manchmal fällt es mir schwer, alles zu glauben, was mein Kind mir erzählt.	☐ 5	☐ 4	☐ 3	☐ 2	☐ 1
3.	Mein Kind ist immer eine guter Zuhörer.	☐ 5	☐ 4	☐ 3	☐ 2	☐ 1
4.	Manchmal traue ich mich nicht, meinem Kind zu sagen, was ich möchte.	☐ 5	☐ 4	☐ 3	☐ 2	☐ 1
5.	Mein Kind neigt dazu, mir Dinge zu sagen, die besser ungesagt bleiben sollten.	☐ 5	☐ 4	☐ 3	☐ 2	☐ 1
6.	Mein Kind weiß, wie ich mich fühle, ohne mich zu fragen.	☐ 5	☐ 4	☐ 3	☐ 2	☐ 1
7.	Ich bin sehr zufrieden damit, wie mein Kind und ich miteinander sprechen.	☐ 5	☐ 4	☐ 3	☐ 2	☐ 1
8.	Wenn ich in Schwierigkeiten wäre, könnte ich das meinem Kind erzählen.	☐ 5	☐ 4	☐ 3	☐ 2	☐ 1
9.	Ich zeige meinem Kind offen Zuneigung.	☐ 5	☐ 4	☐ 3	☐ 2	☐ 1
10.	Wenn wir ein Problem miteinander haben, strafe ich mein Kind oft mit Schweigen.	☐ 5	☐ 4	☐ 3	☐ 2	☐ 1
11.	Ich bin vorsichtig damit, was ich zu meinem Kind sage.	☐ 5	☐ 4	☐ 3	☐ 2	☐ 1
12.	Wenn ich mit meinem Kind spreche, neige ich dazu Dinge zu sagen, die besser ungesagt bleiben sollten.	☐ 5	☐ 4	☐ 3	☐ 2	☐ 1
13.	Wenn ich Fragen stelle, bekomme ich ehrliche Antworten von meinem Kind.	☐ 5	☐ 4	☐ 3	☐ 2	☐ 1
14.	Mein Kind versucht meine Sichtweise zu verstehen.	☐ 5	☐ 4	☐ 3	☐ 2	☐ 1
15.	Ich vermeide es, manche Themen mit meinem Kind zu besprechen.	☐ 5	☐ 4	☐ 3	☐ 2	☐ 1
16.	Es fällt mir leicht, Probleme mit meinem Kind zu besprechen.	☐ 5	☐ 4	☐ 3	☐ 2	☐ 1
17.	Es fällt mir sehr leicht, gegenüber meinem Kind alle meine Gefühle aufrichtig auszudrücken.	☐ 5	☐ 4	☐ 3	☐ 2	☐ 1
18.	Mein Kind stört/nervt mich.	☐ 5	☐ 4	☐ 3	☐ 2	☐ 1
19.	Mein Kind beleidigt mich manchmal, wenn es wütend auf mich ist.	☐ 5	☐ 4	☐ 3	☐ 2	☐ 1
20.	Ich glaube ich kann meinem Kind nicht sagen, was ich wirklich über manche Dinge denke.	☐ 5	☐ 4	☐ 3	☐ 2	☐ 1

Scoring instructions for the PACS-D

1 Compute subscales:

1.1 Subscale communication openness: Sum up scores of items 1, 3, 6, 7, 8, 9, 13, 14, 16, 17 (If there are up to four missing items, sum up scores of answered items, divide by number of answered items, multiply by ten, and round to integer numbers).

1.2 Subscale communication problems: Sum up scores of items 2, 4, 5, 10, 11, 12, 15, 18, 19, 20 (If there are up to four missing items, sum up scores of answered items, divide by number of answered items, multiply by ten, and round to integer numbers). *Recoding(!)*: Substract result from 60.

2 Compute total scale: Sum up scores of communication openness and communication problems subscales.

3 Interpretation: Subscales have a range from 10 to 50. Higher values indicate more openness and less problems, respectively. The total scale has a range from 20 to 100. Higher values indicate higher parent-child communication quality.

Recommendation for a short form:

Items 7, 14, 16 from the open communication subscale and items 4, 12 and 19 from the problem communication subscale.
