# Supplementary material for: Psychometric evaluation of the german version of the parent-adolescent communication scale
Source: Eur Child Adolesc Psychiatry. 2024 Aug 7;34(3):1097–109. doi: 10.1007/s00787-024-02541-4 (PMC11909066; doi:10.1007/s00787-024-02541-4)
Supplement: Supplementary file 3 — Supplementary file3 (DOCX 24 KB) [file 787_2024_2541_MOESM3_ESM.docx]

|  | **Parents** | | | | | **Adolescents** | | | |
| --- | --- | --- | --- | --- | --- | --- | --- | --- | --- |
|  | Population sample  (N = 1041) | | | CAP  (N = 94) | PS  (N = 53) | Population sample  (N = 1028) | CAP  (N = 67) | | PS  (N = 36) |
|  | Overall | Towards daughter | Towards son | Overall | Overall | Overall | Towards mother | Towards father | Overall |
|  | *M* (*SD*) | *M* (*SD*) | *M* (*SD*) | *M* (*SD*) | *M* (*SD*) | *M* (*SD*) | *M* (*SD*) | *M* (*SD*) | *M* (*SD*) |
| Open communication | 39.9 (6.4) | 40.3 (7.6) | 39.6 (6.6) |  |  | 41.6 (7.0) | 36.4 (8.7) | 33.4 (8.9) | 42.8 (5.6) |
| Male | 41.0 (6.8) |  |  | 38.1 (5.6) | 41.0 (7.1) | 41.4 (7.0) | 38.7 (6.4) | 35.0 (8.8) |  |
| Female | 42.0 (7.1) |  |  | 38.7 (5.8) | 40.3 (6.4) | 41.8 (7.0) | 35.9 (9.4) | 32.9 (9.0) |  |
| Problem communication | 38.4 (7.3) | 38.8 (6.9) | 38.1 (7.6) |  |  | 37.6 (7.9) | 33.2 (7.9) | 32.8 (9.1) | 38.1 (6.1) |
| Male | 36.8 (8.1) |  |  | 39.1 (6.8) | 41.0 (5.1) | 37.6 (7.9) | 33.9 (7.3) | 33.5 (8.4) |  |
| Female | 38.1 (7.8) |  |  | 39.0 (5.2) | 41.5 (4.6) | 37.6 (7.9) | 34.1 (7.7) | 33.4 (8.9) |  |
| Total scale | 78.3 (11.7) | 79.0 (11.3) | 77.6 (12.1) |  |  | 79.2 (13.2) |  |  | 80.8 (10.0) |
| Male | 77.9 (13.0) |  |  | 77.2 (9.5) | 82.6 (9.6) | 78.9 (13.3) | 72.7 (10.7) | 68.3 (14.2) |  |
| Female | 80.1 (13.4) |  |  | 77.7 (9.8) | 81.3 (9.4) | 79.4 (13.2) | 69.9 (16.2) | 66.3 (17.2) |  |
|  | | | | | | | | | |

**Supplementary Table 1.** Norm values of the Parent-Adolescent Communication Scale, German translation.
